# Distribution and prognostic implications of right and left ventricular systolic dysfunction in wild-type transthyretin amyloid cardiomyopathy

**DOI:** 10.1007/s10554-026-03614-y

**Published:** 2026-01-19

**Authors:** Ali Hussein Jaber Mejren, Bertil Ladefoged, Anders Lehmann Dahl Pedersen, Tor S. Clemmensen, Marish I. F. J. Oerlemans, Sie Kronborg Fensman, Henrik Vase, Mads J. Andersen, Steen Hvitfeldt Poulsen

**Affiliations:** 1 Department of Cardiology, Aarhus University Hospital, Aarhus, Denmark; 2 Institute of Health, Aarhus University, Aarhus, Denmark; 3 Department of Cardiology, Division of Heart and Lungs, University Medical Center Utrecht, Utrecht, The Netherlands; 4 Member of the European Reference Network for rare, low prevalence and complex diseases of the heart (ERN GUARD-Heart), Amsterdam, The Netherlands

**Keywords:** Wild-type transthyretin amyloid cardiomyopathy, Cardiac amyloidosis, Echocardiography, Biventricular dysfunction

## Abstract

We aimed to characterize the prevalence and severity of left ventricular (LV) and right ventricular (RV) systolic dysfunction and their prognostic implications in wild-type transthyretin amyloid cardiomyopathy (ATTRwt-CM). We enrolled 256 consecutive contemporary ATTRwt-CM patients. LV systolic function was assessed by LV ejection fraction (LVEF), global longitudinal strain (LV-GLS), and stroke volume index (SVi), while RV systolic function was assessed using Tricuspid Annular Plane Systolic Excursion (TAPSE). At ATTRwt-CM diagnosis time, median LVEF was 47.5% (Q1-Q3: 40.7%-54.3%). Preserved LVEF (≥ 50%) was observed in 43.4% of patients, mildly-reduced LVEF (40–49%) in 34.4%, and reduced LVEF (< 40%) in 22.3%. LV-GLS (absolute value: 11.9 ± 3.3%) and SVi (28.6 mL/m²; Q1–Q3: 23.3–34.4) were moderately reduced. Among patients with preserved LVEF, 90% had impaired LV-GLS and 57% reduced SVi. Impaired LVEF (< 50%) was associated with higher all-cause mortality compared with preserved LVEF (adjusted hazard ratio [aHR] 2.0; 95% CI: 1.26–3.17). TAPSE was 17.7 ± 5.1 mm and demonstrated an independent association with mortality (aHR 0.92; 95% CI: 0.88–0.96). One-third of patients had preserved LVEF and TAPSE (≥ 18 mm), showing the most favorable prognosis compared with patients with impaired LVEF, TAPSE, or both. LV and RV systolic dysfunction are common at the time of ATTRwt-CM diagnosis and span a broad spectrum of severity. LV-GLS and SVi are frequently impaired despite preserved LVEF, highlighting the need for a multiparametric assessment of LV function. Incorporating TAPSE for RV evaluation may further refine risk stratification, particularly when LVEF is preserved.

**Graphical Abstract:**

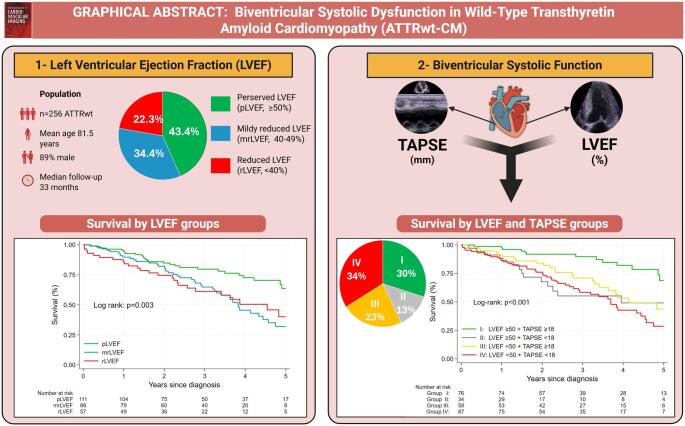

## Introduction

Wild-type transthyretin amyloid cardiomyopathy (ATTRwt-CM) is the most prevalent form of ATTR-cardiomyopathy (ATTR-CM), characterized by progressive myocardial infiltration by amyloid fibrils, leading to increased wall thickness, stiffness, and cardiac dysfunction [1, 2]. Recognition of ATTRwt-CM has increased substantially in recent years, driven by advances in imaging and greater clinical awareness, with therapeutic advances further supporting recognition [3]. Cardiac amyloidosis—including ATTRwt-CM—has traditionally been considered primarily as a form of diastolic heart failure (HF), supported by findings that ATTR-CM is present in up to 13% of patients with HF and preserved left ventricular (LV) ejection fraction (EF) [4]. Thus, current guidelines from the European Society of Cardiology recommend considering ATTR-CM in patients with HF, increased wall thickness, and preserved left ventricular ejection fraction (pLVEF) [5]. However, emerging evidence suggests that the LVEF spectrum in ATTR-CM is wider than previously anticipated, with up to 60% of patients demonstrating impaired LVEF (< 50%) [6–9]. This supports adopting a diagnostic approach irrespective of LVEF in patients suspected of ATTR-CM.

Amyloid deposition involves not only the left but also the right heart chambers, with possible impairment of right ventricular (RV) and atrial function [10]. Yet, the extent and prognostic impact of right-sided dysfunction in ATTR-CM remain poorly defined. This study aims to characterize LV and RV systolic function by echocardiography and to assess the prognostic impact of biventricular systolic dysfunction in a large, contemporary cohort of ATTRwt-CM patients.

## Methods and materials

This single-center cohort study comprised 256 consecutive ATTRwt-CM patients diagnosed from January 1st, 2016, to December 31st, 2023, at the Department of Cardiology at Aarhus University Hospital, Denmark.

Patients were included if they had a confirmed diagnosis of ATTRwt-CM, established by either positive 3,3-Diphosphono-1,2-Propanodicarboxylic Acid (DPD) scintigraphy (Perugini grade 2–3), evidence of ATTR on endomyocardial biopsy, or both. AL amyloidosis was excluded using guideline-recommended diagnostic tests [5]. Variant ATTR-CM was excluded by genetic testing. The time of diagnosis was defined as the date of DPD scintigraphy or the date of endomyocardial biopsy if the latter was performed to confirm the diagnosis.

The disease stage definitions from the National Amyloidosis Centre (NAC), London, were used to categorize disease severity [11].

### Data collection

Clinical characteristics, biomarkers, electrocardiograms (ECGs), and baseline comprehensive transthoracic echocardiography were collected prospectively at the time of diagnosis and recorded in a dedicated ATTRwt-CM database. Echocardiographic analyses for this study were performed offline by a single experienced investigator (AHJM) to ensure consistency. ECG, echocardiographic, and mortality data were retrieved from local electronic health records, the regional imaging system, and the Danish Civil Registry, respectively.

### Echocardiography

Transthoracic echocardiography was performed according to current guidelines using a Vivid E95 system (GE Healthcare, Horten, Norway), and data were analyzed offline with EchoPAC software (version 204, GE Healthcare) [12]. LVEF was measured using Simpson’s biplane method. Patients were categorized according to baseline LVEF into pLVEF for LVEF ≥ 50%, mildly-reduced LVEF (mrLVEF) for LVEF 40–49%, and reduced LVEF (rLVEF) for LVEF < 40%.

Stroke volume (SV) was calculated as the product of the LV outflow tract area and velocity-time integral by pulsed-wave Doppler, and it was indexed to body surface area (BSA) to obtain the SV index (SVi). The LV global longitudinal strain (LV-GLS) was obtained using an automated speckle-tracking function from standard two-dimensional four-, two-, and three-chamber apical projections with a frame rate > 55 frames/second. Manual adjustments were performed when necessary to optimize tracking. LV-GLS is reported as absolute numerical values. In patients with atrial fibrillation, triplane imaging using a 4-dimensional probe was used for LV-GLS measurements. Left atrial (LA) volume was measured using the biplane disk summation method [12]. However, a single plane (four-chamber view) was used in 14% of the patients due to the suboptimal two-chamber atrial view. LA volume index (LAVi) was obtained by indexing LA volume to BSA. Right atrial (RA) volume was measured in a RA-focused four-chamber view, using the single-plane disk summation method and indexed to BSA to calculate RA volume index (RAVi). Tricuspid annular plane systolic excursion (TAPSE) was measured at the lateral tricuspid annulus using M-mode. RV free wall thickness was measured in the subcostal long-axis view at the basal RV segment, and the inferior vena cava was evaluated from the substernal two-dimensional view. RV systolic pressure was estimated by adding the pressure gradient across the tricuspid valve, calculated from the tricuspid regurgitation jet using the simplified Bernoulli equation, to the estimated RA pressure, which was derived from the inferior vena cava diameter and collapsibility [12].

The intra- and inter-observer reproducibility of LVEF, LV-GLS, and TAPSE was evaluated in a subset of 20 echocardiographic studies selected to ensure adequate image quality and a representative range of LVEF values. Measurements were repeated by the primary observer (AHJM) and independently by a second experienced observer (SKF), both blinded to each other’s results and to the original analyses. Each observer reviewed the studies from the same examination date and selected the image(s) or loop(s) they considered most suitable for each measurement. Reproducibility was assessed using intraclass correlation coefficients (ICC) and the absolute difference (SD) between paired measurements.

### Follow-up

All patients were followed from diagnosis until death, five years of follow-up, or until the censoring date of 1st November 2024. No patients were lost during follow-up.

### Statistical analysis

Continuous data are presented as mean ± SD for normally distributed variables or median and interquartile range (Q1–Q3) for non-normally distributed variables. Categorical variables are reported as numbers and percentages. Group differences were compared using multiple linear regression for normally distributed continuous variables or the Kruskal-Wallis test for non-normally distributed variables. Categorical variables were compared using the chi-squared test. Linear regression assumptions were checked using residual plots. Missing data for key echocardiographic parameters were low (LV-GLS 3.9%, SVi 9.4%, TAPSE 0.4%, RAVi 0.4%), with no missing values for LVEF or LAVi. The missingness pattern for SVi was consistent with data missing completely at random, whereas LV-GLS values were missing at random. Given these patterns and the small proportion of missing data, complete-case analysis was applied.

Survival analyses were conducted using Kaplan-Meier (KM) estimates, with all-cause mortality as the outcome and group differences assessed by the log-rank test. Hazard ratio (HR), 95% confidence interval (CI), and two-sided p-value were determined using Cox proportional-hazards regression models. Both crude HR and adjusted HR (aHR) were reported, with adjustment for clinically relevant confounders. Models’ assumptions were checked using log-log plots and predicted survival plots for categorical variables, and by Schoenfeld residuals test for continuous variables. The linearity of continuous covariates was evaluated using restricted cubic spline smoothers. We performed two sensitivity analyses to test the robustness of our results; in the first analysis, we excluded patients who were randomized into clinical trials during follow-up from the Cox Regression analysis. In the second analysis, we included atrial fibrillation and/or atrial flutter status as an additional covariate in the multivariable Cox models for LVEF, LV-GLS, SVi, and TAPSE. Two-sided tests were used for all analyses, and *p* < 0.05 was considered significant. The data were analyzed using STATA (STATA/SE 19, StataCorp LLC, College Station, TX, USA).

## Results

### Overall clinical and echocardiographic characteristics of the ATTRwt-CM population

The baseline characteristics of the 256 enrolled ATTRwt-CM patients are demonstrated in Table 1. They were predominantly male (89%), Caucasians (non-Caucasian < 5 patients), and with a mean age at diagnosis of 81.5 ± 5.9 years. Most patients were in New York Heart Association (NYHA) class I-II (77%). At the time of diagnosis, 79% of the patients were receiving diuretic therapy. No patient received specific ATTR-modifying therapy because of the lack of regulatory approval during the study period. However, during the follow-up period, 61 patients (24%) participated in one of the following double-blinded, randomized controlled clinical trials testing ATTR-modifying therapy: HELIOS-B (vutrisiran), APOLLO-B (patisiran), or ATTRibute-CM (acoramidis). The participation rate in clinical trials did not differ significantly among LVEF categories (*p* = 0.27). Overall, 56% of the patients had a history of atrial fibrillation and/or flutter prior to ATTRwt-CM diagnosis, and 35% had ongoing atrial fibrillation and/or flutter at the time of diagnosis.

**Table 1:** Clinical characteristics by LVEF categories

	All (*n* = 256)	pLVEF (*n* = 111)	mrLVEF (*n* = 88)	rLVEF (*n* = 57)	*p*-value
Age, y	81.5 ± 5.9	80.8 ± 6.1	82.0 ± 5.3	82.0 ± 6.3	0.270
Male sex	228 (89)	97 (87)	79 (90)	52 (91)	0.726
Body mass index, kg/m^2^	26 ± 3.4	26 ± 3.5	26 ± 3.4	26 ± 3.2	0.411
Heart rate, beat/min	72 (63–81)	70 (63–80)	70 (61–77)	80 (71–88)	< 0.001
Systolic blood pressure, mmHg	136 ± 20	140 ± 20	136 ± 19	132 ± 21	0.071
Diastolic blood pressure, mmHg	80 ± 11	80 ± 10	79 ± 11	81 ± 13	0.466
NYHA I/II	78 (31) /120 (47)	47 (42) / 48 (43)	18 (21) / 40 (46)	13 (23) / 32 (56)	0.003
III/IV	55 (22) / 3 (1)	15 (14) / 1 (1)	28 (32) / 2 (2)	12 (21) / 0 (0)	
NAC stage: 1	140 (55)	74 (67)	42 (48)	24 (42)	0.008
2	77 (30)	28 (25)	29 (33)	20 (35)	
3	39 (15)	9 (8)	17 (19)	13 (23)	
Diabetes mellitus	40 (16)	11 (10)	15 (17)	14 (25)	0.042
Ischemic heart disease	49 (19)	25 (23)	15 (17)	9 (16)	0.467
Hypertension	160 (63)	67 (61)	53 (61)	40 (70)	0.443
COPD	34 (14)	12 (11)	13 (15)	9 (16)	0.597
Atrial fibrillation/flutter	142 (56)	53 (48)	51 (58)	38 (67)	0.055
Pacemaker	58 (23)	16 (14)	21 (24)	21 (37)	0.004
Carpal tunnel syndrome	92 (36)	37 (33)	33 (38)	22 (39)	0.743
Lumbar spinal stenosis	29 (11)	15 (14)	9 (10)	5 (9)	0.605
eGFR (mL/min/1,73 m²)	62 (45–78)	65 (48–81)	60 (41–75)	62 (43–77)	0.029
NT-proBNP (ng/L)	2217 (1235–3995)	1577 (665–2736)	2446 (1572–4228)	3125 (2214–5665)	< 0.001
Troponin I (ng/L) (*n* = 193) *	52 (30–88)	39 (26–63)	67 (44–112)	58 (36–89)	0.005
Betablocker	104 (41)	43 (39)	34 (39)	27 (47)	0.517
Diuretics	201 (79)	73 (66)	73 (84)	55 (97)	< 0.001

Values are mean ± SD, median (Q1-Q3) or n (%). Comparisons between left ventricular ejection fraction (LVEF) categories were performed using multiple linear regression or Kruskal-Wallis test for continuous variables, and chi-squared test for categorical variables, as appropriate

*Parameters with missing data > 10%. n = number of patients with available data

pLVEF = preserved LVEF (EF ≥ 50%), mrLVEF = mildly-reduced LVEF (EF 40–49%), rLVEF = reduced LVEF (EF < 40%); NYHA = New York Heart Association; NAC = National Amyloidosis Centre, COPD = chronic obstructive lung disease; eGFR = estimated glomerular filtration rate. NT-proBNP = N-terminal pro-B-type natriuretic peptide

**Fig. 1:**
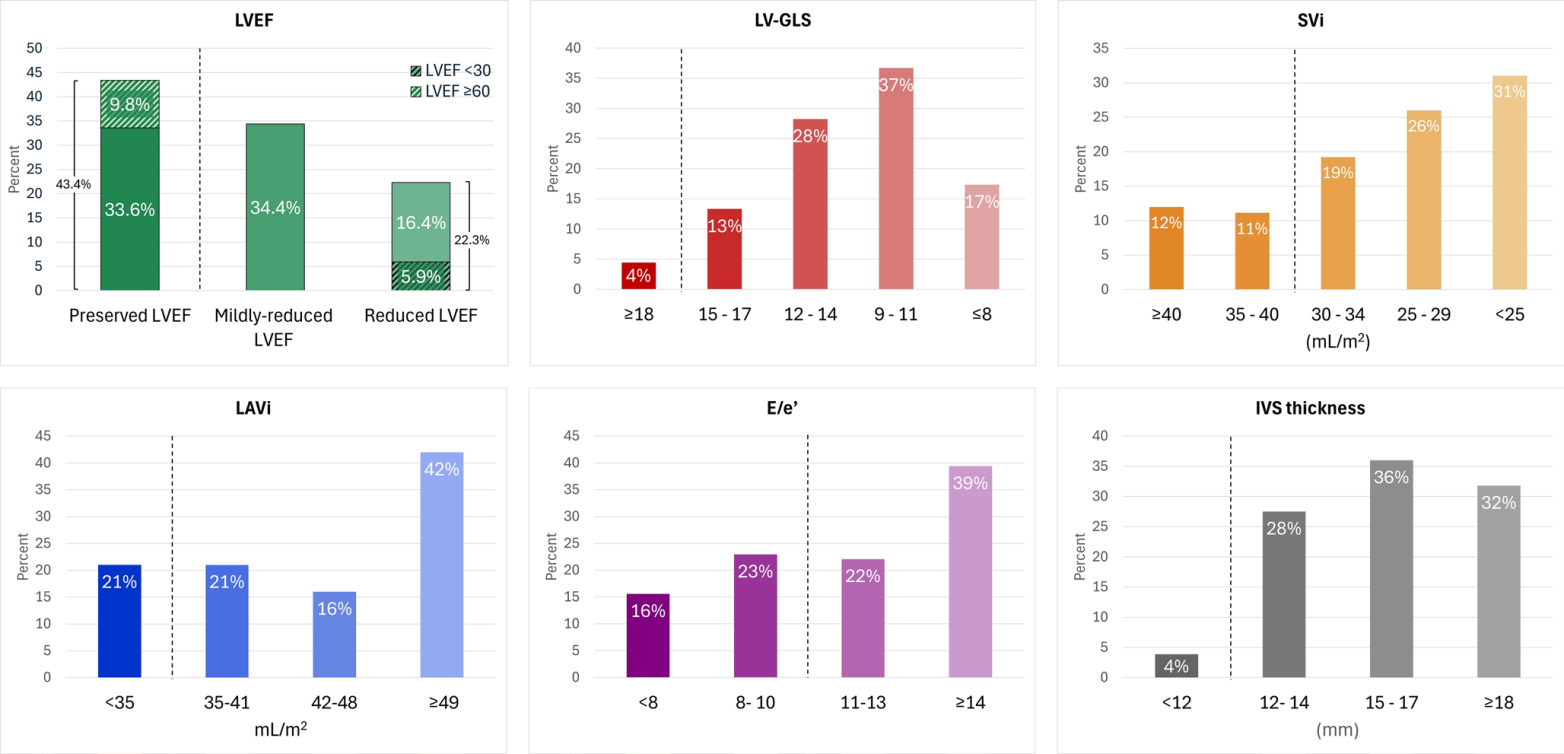
Distribution of left-sided structural and functional echocardiographic parameters. Dotted lines indicate commonly accepted thresholds for abnormal values. LVEF = left ventricular ejection fraction; LV-GLS = Left ventricular global longitudinal strain; SVi = Stroke volume index; LAVi = Left atrial volume index; E/eʹ= ratio of early mitral inflow (E) to lateral eʹ; IVS = Interventricular septal thickness

Table 2 presents the distribution of the echocardiographic parameters for the entire cohort. Overall, LVEF was mildly reduced, with a median of 47.5% (Q1–Q3: 40.7–54.3); SVi was reduced with a median of 28.6 mL/m^2^ (Q1–Q3: 23.3–34.2), and LV-GLS was moderately reduced with a mean of 11.9 ± 3.3%. The interventricular septum was moderately thickened with a mean of 16.1 ± 3.0 mm. Approximately one-third of patients lacked severe LV hypertrophy, with interventricular septal thickness ≤ 14 mm. The LA was enlarged with a median LAVi of 45.1 mL/m^2^ (Q1–Q3: 36.5–56.7). Lateral E/e′ was mildly elevated with a median of 12.4 (Q1–Q3: 9.3–15.8); however, 39% of patients had values ≥ 14. The overall distributions of left-sided echocardiographic parameters are displayed in Fig. 1.

**Table 2:** Left and right echocardiographic parameters by LVEF categories

	All (*n* = 256)	pLVEF (*n* = 111)	mrLVEF (*n* = 88)	rLVEF (*n* = 57)	*p*-value
Sinus rhythm at baseline	166 (65)	81 (73)	53 (60)	32 (57)	0.067
LVEF, %	47.5 (40.7–54.3)	55.8 (51.5–59.4)	44.1 (42.1–47.0)	35.1 (29.9–37.8)	
LV EDV, mL	98.8 ± 31.0	91.2 ± 26.2	101.0 ± 32.1	110.0 ± 34.1	< 0.001
LV ESV, mL	53.1 ± 22.2	40.2 ± 12.9	56.1 ± 18.3	73.7 ± 25.0	< 0.001
LV-GLS, %	11.9 ± 3.3	14.0 ± 3.0	11.2 ± 2.0	8.5 ± 2.0	< 0.001
SVi, mL/m²	28.6 (23.3–34.2)	33.5 (28.4–39.8)	26.8 (23.3–30.4)	24.2 (20-28.7)	< 0.001
CI, L/min/m²	2.0 (1.6–2.5)	2.3 (1.9–2.9)	1.8 (1.6–2.1)	1.9 (1.5–2.1)	< 0.001
IVS, mm	16.1 ± 3.0	15.7 ± 2.9	16.3 ± 3.0	16.7 ± 3.1	0.071
PW, mm	13.7 ± 3.1	13.0 ± 2.7	14.1 ± 3.2	14.5 ± 3.3	0.003
LVMi, g/m²	144 ± 43	134 ± 41	147 ± 45	161 ± 37	< 0.001
LAVi, mL/m²	45.1 (36.5–56.7)	43.4 (33.3–55.1)	46.5 (37.6–57.0)	48.4 (40.2–57.5)	0.034
Lateral *e*ʹ, cm/s	6 (5–8)	7 (6–9)	6 (5–8)	6 (4–8)	< 0.001
Deceleration time, ms	182 (152–228)	196 (163–243)	171 (145–213)	166 (145–188)	0.042
*E/A* ratio (*n* = 166)*	1.37 (0.86–2.26)	1.23 (0.81–2.06)	1.45 (1.0-2.1)	1.69 (0.95–2.78)	0.097
*E*/*e*ʹ lateral	12.4 (9.3–15.8)	11.2 (8.5 14.7)	12.8 (9.9 16.8)	13.8 (9.5 19.9)	0.002
Mitral insufficiency (> mild)	16 (6.35)	6 (5.45)	6 (6.9)	4 (7.27)	0.873
Aortic stenosis^†^	36 (16)	17 (17)	12 (15)	7 (14)	0.949
TAPSE, mm	17.7 ± 5.1	19.7 ± 4.8	17.3 ± 5.0	14.6 ± 4.0	< 0.001
RAVi, mL/m²	38.3 (27.8–51.3)	31.6 (23.7–44.6)	41.3 (30.6–57.4)	40.2 (33.2–55.4)	0.002
Tricuspid regurgitation gradient, mmHg (*n* = 184)*^‡^	30.8 ± 9.6	29.7 ± 9.9	30.0 ± 9.2	33.6 ± 9.2	0.075
RV systolic pressure, mmHg (*n* = 168)	36.9 ± 11.2	35.1 ± 10.8	35.8 ± 11.4	41.5 ± 10.5	0.012
RV FWT, mm (*n* = 200)*	7.0 ± 1.6	6.8 ± 1.7	7.2 ± 1.7	6.9 ± 1.3	0.329

Values are mean ± SD, median (Q1-Q3) or n (%). Comparisons between LVEF categories were performed using multiple linear regression or Kruskal-Wallis test for continuous variables, and chi-squared test for categorical variables, as appropriate

*Parameters with missing data > 10%. n = number of patients with available data

^†^Aortic stenosis is defined as aortic valve mean gradient ≥ 20 mmHg

^‡^Tricuspid regurgitation jet was detectable in only 72% of the patients

LV = Left ventricle; LVEF = LV ejection fraction; pLVEF = preserved LVEF (EF ≥ 50%), mrLVEF = mildly-reduced LVEF (EF 40–49%), rLVEF = reduced LVEF (EF < 40%); LV-EDV = LV end-diastolic volume; LV-ESV = LV end-systolic volume; LV-GLS = LV global longitudinal strain; SVi = Stroke volume index; CI = Cardiac index; IVS = Interventricular septal thickness; PW = Posterior wall thickness; LVMi = LV mass index; LAVi = Left atrial volume index; E velocity = Peak early diastolic mitral inflow velocity; lateral eʹ= Peak early diastolic velocity of the lateral mitral annulus; E/A ratio = ratio of early (E) to late (A) diastolic mitral inflow velocities; TAPSE = Tricuspid annular plane systolic excursion; RAVi = Right atrial volume index; RV = Right ventricle; RV FWT = Right ventricular free wall thickness; IVC = Inferior vena cava

**Fig. 2:**
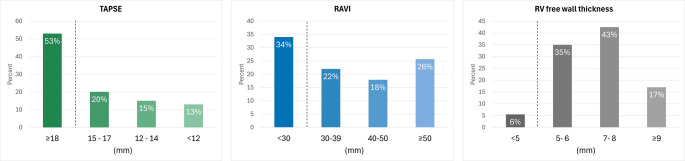
Distribution of right-sided structural and functional echocardiographic parameters. Dotted lines indicate commonly accepted thresholds for abnormal values. TAPSE = Tricuspid annular plane systolic excursion; RAVi = Right atrial volume index; RV FWT = Right ventricular free wall

The RV free wall was thickened with a mean of 7.0 ± 1.6 mm, median RAVi was 38.3 mL/m^2^ (Q1–Q3: 27.8–51.3), and mean TAPSE was 17.7 ± 5.1 mm. The distributions of RAVi, RV free wall thickness, and TAPSE are displayed in Fig. 2.

### Clinical and echocardiographic characteristics across LVEF categories

Overall, 43.4% of the patients had pLVEF at the time of diagnosis, 34.4% presented with mrLVEF, and 22.3% had rLVEF. Table 1 outlines the clinical characteristics, and Table 2 shows the echocardiographic parameters across the LVEF categories. Patients in the lower LVEF categories tended to have more advanced NAC disease stage and were more symptomatic as compared to patients with pLVEF. A significant increase in LAVi, E/e′ ratio and LVMi was observed across the decreasing LVEF categories, accompanied by a corresponding decrease in LV-GLS and SVi.

**Fig. 3:**
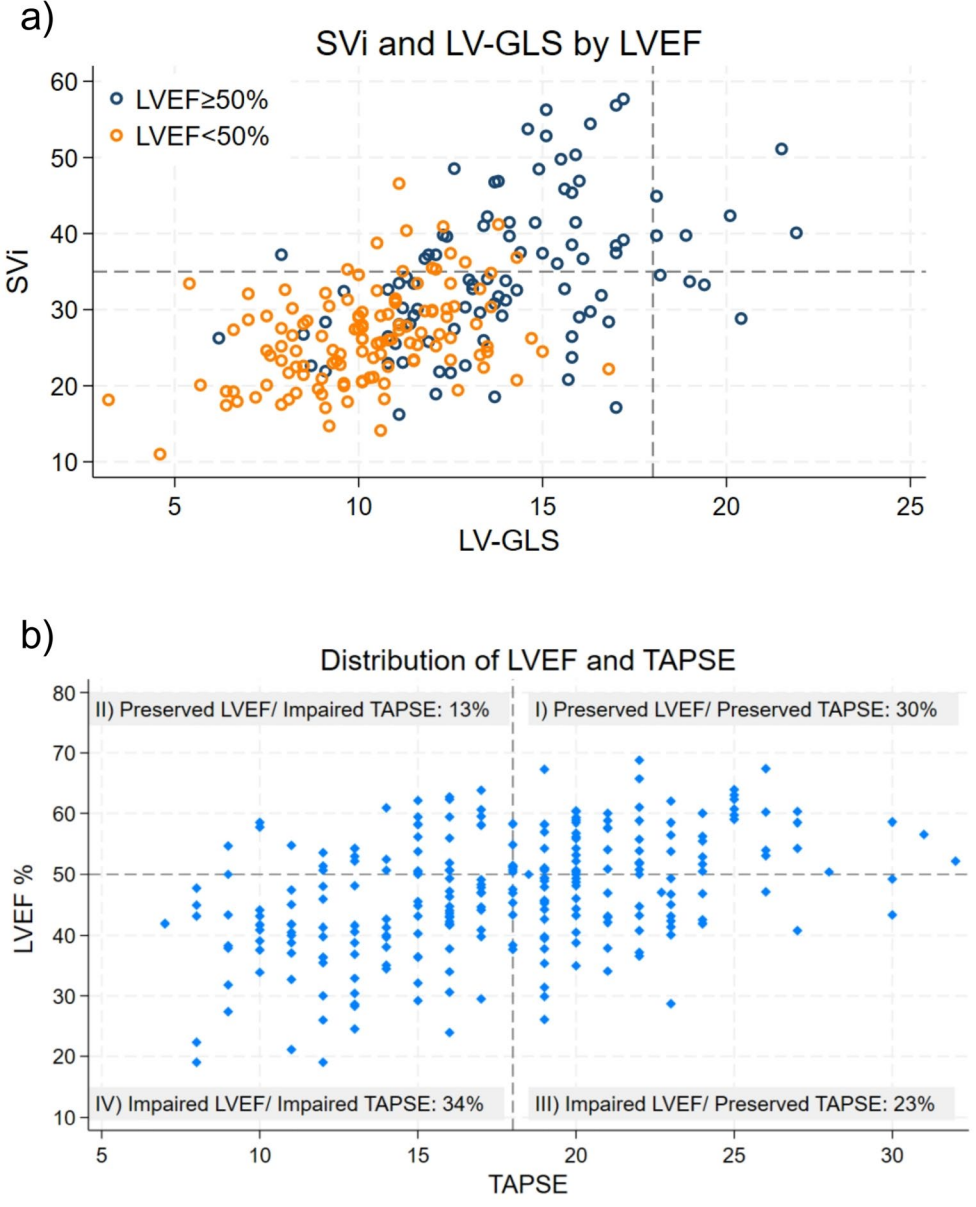
**a**: Scatter plot showing the relationship between SVi and LV-GLS stratified by LVEF ≥ 50% or < 50%. Dotted lines indicate commonly accepted thresholds for abnormal values. **b**: Scatter plot showing the relationship between LVEF and TAPSE. Dotted lines indicate commonly accepted thresholds for abnormal values. Data was grouped into 4 groups: group (I) preserved LVEF (≥ 50% ) and preserved TAPSE (≥ 18 mm); group (II) preserved LVEF and impaired TAPSE (< 18 mm); group (III) impaired LVEF (< 50%) and preserved TAPSE; and group (IV) impaired LVEF (< 50%) and impaired TAPSE. LVEF = left ventricular ejection fraction; LV-GLS = Left ventricular global longitudinal strain; SVi = Stroke volume index; TAPSE = Tricuspid annular plane systolic excursion

In the pLVEF category, median LVEF was 55.8% (Q1–Q3: 51.5–59.4); however, a reduced SVi (< 35 mL/m^2^) was present in 57% of the patients, and 90% had reduced LV-GLS (< 18%). The relationship between LV-GLS and SVi, stratified by LVEF ≥ 50% or < 50%, is demonstrated in Fig. 3A.

RV systolic function evaluated by TAPSE decreased significantly across declining LVEF categories, while RAVi increased significantly. Figure 3b shows the relationship between LVEF and TAPSE, dividing the cohort into four groups based on preserved (≥ 50%) or impaired LVEF (< 50%) and preserved (≥ 18 mm) or impaired (< 18 mm) TAPSE. Group II (pLVEF and impaired TAPSE) constitutes 13% of the cohort. Compared to group I ( pLVEF and preserved TAPSE), group II had lower LV-GLS [(13.0±3.17%) vs. (14.4 ± 2.76%), *p* = 0.25], lower SVi [29.2 mL/m^2^ (Q1-Q3: 23.1– 39.6) vs. 34.2 mL/m^2^ (Q1-Q3: 30.2–40.1); *p* = 0.016], and a higher prevalence of atrial fibrillation/flutter (67.6% vs. 39.5%; *p* = 0.007). RV wall thickness was numerically larger in group II, though statistically insignificant [(6.63 ± 1.75 mm) vs. (7.27 ± 1.56 mm); *p* = 0.123]. Likewise, there were no significant differences in lateral E/e′, LAVi, or RAVi.

Alternative RV measures such as fractional area change, RV strain, and S′ velocity were not consistently available. In the subset with available S′ data (42% of the cohort), TAPSE and S′ showed a moderate correlation (*r* = 0.57), supporting the use of TAPSE as a marker of RV systolic function in this cohort.

### Reproducibility

Intra-observer reproducibility was good, with ICCs of 0.91 for LVEF, 0.93 for LV-GLS, and 0.88 for TAPSE. The absolute differences (mean ± SD) were 2.6 ± 4.2% points for LVEF, − 0.5 ± 1.2% points for LV-GLS, and 0.75 ± 2.3 mm for TAPSE. Inter-observer reproducibility was similarly good, with ICCs of 0.89 for LVEF, 0.87 for LV-GLS, and 0.89 for TAPSE. Corresponding absolute differences were 2.1 ± 5.3% points for LVEF, − 1.0 ± 1.6% points for LV-GLS, and 0.4 ± 2.2 mm for TAPSE. Overall, inter- and intra-observer agreement was consistent with previously reported reproducibility for these parameters [13–15].

### Survival analysis

A total of 94 patients (37%) died during a median follow-up of 33 months (Q1–Q3: 20–49). The survival rates after diagnosis were 91% (95% CI: 87–94) at one year, 82% (95% CI: 76–86) at two years, 70% (95% CI: 64–76) at three years, 57% (95% CI: 50–64) at four years, and 47% (95% CI: 38–55) at five years.

**Fig. 4:**
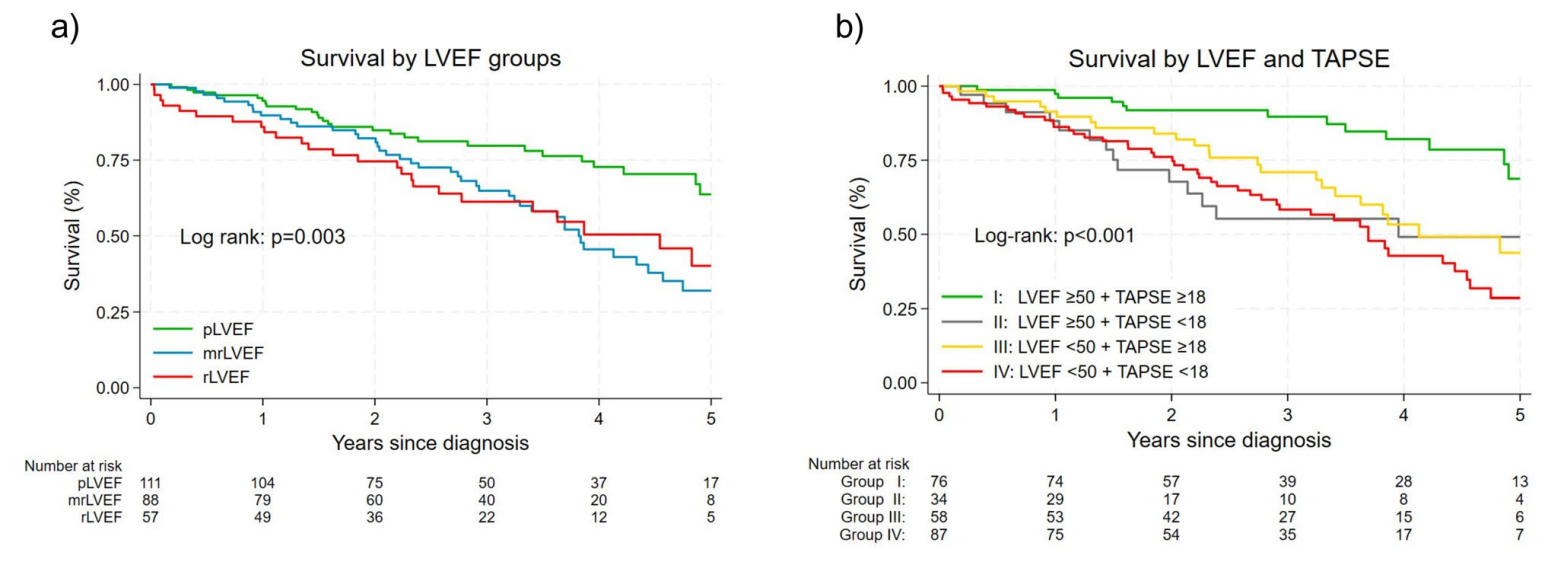
**a**: Kaplan–Meier survival curves by LVEF categories. **b**: Kaplan–Meier survival curves by LVEF (≥ 50% vs. <50%) and TAPSE (≥ 18 mm. vs. < 18 mm) groups. LVEF = left ventricular ejection fraction; pLVEF = preserved LVEF (EF ≥ 50%), mrLVEF = mildly-reduced LVEF (EF 40–49%), rLVEF = reduced LVEF (EF < 40%); TAPSE = Tricuspid annular plane systolic excursion

LVEF was associated with mortality with an aHR of 0.97 (95% CI: 0.95–0.99, *p* = 0.01), indicating that a 1% increase in LVEF was associated with a 3% decrease in all-cause mortality. KM analysis showed that the pLVEF group had the best prognosis compared to the mrLVEF and rLVEF groups. No difference in survival was observed between the mrLVEF and rLVEF groups (Fig. 4a)*.* Comparing mortality between the impaired LVEF group (i.e. LVEF < 50%) and the pLVEF group revealed an aHR of 2.00, 95% CI: 1.26–3.17, *p* = 0.003. For other LV systolic parameters, LV-GLS was associated with all-cause mortality, as each 1% increase corresponded to a lower risk (aHR 0.89, 95% CI: 0.83–0.96, *p* = 0.002). SVi showed an aHR of 0.98, 95% CI 0.95–1.00, *p* = 0.081). LAVi showed an aHR of 1.01 (95% CI: 1.00–1.02, *p* = 0.105).

Sensitivity analyses excluding patients enrolled in clinical trials and adjusting the Cox models for atrial fibrillation/flutter did not alter the HR estimates, confirming the robustness of the findings.

For the right-sided echocardiographic parameters, both TAPSE and RAVi were associated with all-cause mortality, as RAVi demonstrated an aHR of 1.02 (95% CI: 1.01–1.03, *p* < 0.001), and TAPSE had an aHR of 0.92 (95% CI: 0.88–0.96, *p* < 0.001). Detailed Cox regression analysis results and model descriptions for the left- and right-sided echocardiographic parameters are summarized in Table 3.

**Table 3:** Univariable and multivariable Cox regression analysis of left- and right-side echocardiographic parameters

Parameter	Crude HR (95% CI)	*p*-value	aHR (95% CI)	*p*-value
**Left-side echocardiographic parameters**				
LVEF (%)*^†^	0.97 (0.95–0.99)	0.004	0.97 (0.95–0.99)	0.011
LVEF < 50% vs. LVEF ≥ 50%^†^	2.16 (1.38–3.39)	0.001	2.00 (1.26 − 3.17)	0.003
LV-GLS (%)*^†^	0.89 (0.83–0.96)	0.001	0.89 (0.83–0.96)	0.002
SVi (mL/m²)*^†^	0.98 (0.95–1.00)	0.066	0.98 (0.95–1.00)	0.081
LAVi (mL/m²)*^‡^	1.01 (1.00–1.02)	0.059	1.01 (1.00–1.02)	0.105
**Right-side echocardiographic parameters**				
RAVi (mL/m^2^)*^§^	1.01 (1.01–1.02)	< 0.001	1.02 (1.01–1.03)	< 0.001
TAPSE (mm)*^¶^	0.91 (0.87–0.95)	< 0.001	0.92 (0.88–0.96)	< 0.001

*Per unit increase

^†^Model I: adjusted for age, sex, diabetes mellitus, hypertension, and ischemic heart disease

^‡^Model II: adjusted for age, sex, diabetes mellitus, hypertension, ischemic heart disease, and the presence of atrial fibrillation and/or flutter

^§^Model III: Adjusted for age, sex, atrial fibrillation and/or flutter, pacemaker, and moderate or severe tricuspid regurgitation

^¶^Model IV: Adjusted for age, sex, and previous open-heart surgery

LVEF = left ventricular ejection fraction; LV-GLS = LV global longitudinal strain; SVi = Stroke volume index; LAVi = Left atrial volume index; RAVi = Right atrial volume index; TAPSE = Tricuspid annular plane systolic excursion

Figure 4b illustrates the KM analysis of the cohort stratified into four groups based on preserved (≥ 50%) or impaired LVEF (< 50%) and TAPSE. A significant difference in mortality was observed among the groups (log-rank *p* < 0.001), with group I (preserved LVEF and TAPSE) showing the most favorable prognosis. In contrast, the survival curves of the remaining three groups overlapped, indicating no significant differences in mortality among them.

## Discussion

The main findings of the present study in contemporary ATTRwt-CM patients are as follows: First, the majority of patients had impaired LVEF (< 50%) at the time of diagnosis. Second, despite pLVEF, almost all patients demonstrated impaired LV-GLS, and more than half had reduced SVi. Third, impaired LVEF (< 50%) was associated with a worse prognosis. Fourth, right-sided cardiac dysfunction was common, as half of the patients demonstrated impaired RV systolic function by reduced TAPSE, and two-thirds of patients exhibited RA enlargement, suggesting some degree of restrictive RV filling. Finally, approximately one-third of patients had preserved LVEF and TAPSE and demonstrated the most favorable prognosis compared with those who had either impaired LVEF, impaired TAPSE, or a combination of both.

### LV systolic function

In our ATTRwt-CM cohort, 56% of patients had impaired LVEF (< 50%) at the time of diagnosis, challenging the traditional perception of ATTR-CM as primarily a restrictive cardiomyopathy with pLVEF [16, 17], and aligning with findings from recent studies [6–9]. In addition, one-third of our cohort did not exhibit severe LV hypertrophy, with an interventricular septal thickness of ≤ 14 mm. These findings underscore the need to consider ATTRwt-CM across the entire LVEF spectrum, even in patients with only mildly thickened LV walls.

Other important LV-systolic parameters like LV-GLS and SVi were generally reduced in our cohort, even among patients with pLVEF. This highlights the importance of assessing other LV-systolic parameters in this patient group, as LVEF may underestimate the degree of LV systolic dysfunction.

### LV diastolic function

Progressive myocardial amyloid accumulation increases LV stiffness, leading to elevated diastolic filling pressures both at rest and during exercise, and is accompanied by structural changes such as LA dilatation [18]. LA enlargement can be considered a marker of chronic LV diastolic dysfunction in the absence of significant mitral valve disease. In our cohort, LA was moderately enlarged, and 79% of our cohort had a dilated LA ≥ 35 mL/m^2^. These findings support the general conception that LV diastolic dysfunction is a prominent feature of ATTRwt-CM. However, other factors, such as direct amyloid infiltration of the atrial walls and the presence of atrial fibrillation or flutter, may also contribute to LA enlargement.

Even though the overall median lateral E/e′ ratio was only borderline elevated, 39% of the ATTRwt-CM patients had a markedly elevated lateral E/e′ (≥ 14), which is strongly indicative of diastolic dysfunction [19]. Nonetheless, interpretation of E/e′ ratio in ATTR-CM remains uncertain, as the relationship between E/e′ and LV filling pressure has not, to our knowledge, been specifically validated in this population.

### RV structure and function

While LV systolic and diastolic dysfunction play a central role in the pathophysiology, clinical performance, and prognosis of ATTR-CM, right-sided parameters have been less investigated [20, 21]. Porcari et al. recently showed that diffuse RV-uptake on bone scintigraphy was associated with a poor prognosis in ATTR-CM [22].

In the present study, we noted that 95% of ATTRwt-CM patients had some degree of RV free wall thickening, and the RV longitudinal systolic function by TAPSE was reduced (< 18 mm) in approximately half of the patients. RA enlargement was also a prominent feature, as this was present in two-thirds of the patients. In addition, we observed that 13% of the cohort demonstrated impaired RV systolic function with pLVEF (group II). Compared with group I (pLVEF and preserved TAPSE), group II exhibited lower SVi and LV-GLS, suggesting a degree of LV-systolic dysfunction even in the presence of pLVEF. They also had a higher prevalence of atrial fibrillation/flutter, whereas E/e′ did not differ between the two groups. These findings might suggest backward failure as a likely primary mechanism of RV impairment. Nevertheless, since both groups showed increased RV wall thickness, with a trend for thicker walls in group II, direct RV involvement due to amyloid infiltration cannot be excluded.

### Prognostic implications

In our study, LVEF was significantly associated with all-cause mortality, which is consistent with the findings of previous studies [23, 24]. This suggests that patients with impaired LVEF (< 50%) at the time of diagnosis may have more advanced disease than those with pLVEF, as also reflected by differences in NAC stage and NYHA class observed among pLVEF, mrLVEF, and rLVEF categories in our cohort. Interestingly, all-cause mortality was comparable between rLVEF and mrLVEF categories,, which may indicate that LVEF provides limited additional prognostic value in ATTRwt-CM patients with lower LVEF categories.

Longitudinal strain, performed on isolated 4-chamber views, has been shown to contain prognostic information [23]. In our study, we performed a global strain assessment (GLS), which also demonstrated a significant association with mortality, supporting its role as an important marker of LV systolic function.

Doppler-derived SVi was also frequently reduced in our study. Previous work examining volume-based SVi demonstrated an association with prognosis, with an aHR of 0.97 (95%CI: 0.95–0.99), *p* = 0.004 [23]. In our study, Doppler-derived SVi showed a nearly identical effect estimate and CI (aHR of 0.98 (95%CI: 0.95–1.00, *p* = 0.081). Although the p-value exceeded 0.05, the effect estimate and confidence interval closely align with prior findings, suggesting that our analysis may have been limited by sample size, rather than reflecting a true absence of association.

Both TAPSE and RAVi have individually been shown to provide prognostic information in patients with ATTR-CM [23, 25]. This was confirmed in our cohort, as both were independently associated with all-cause mortality. Furthermore, the KM survival analysis demonstrated that patients with impaired TAPSE despite preserved LVEF experienced a significantly worse prognosis compared with those with both preserved LVEF and TAPSE. This finding highlights the potential role of TAPSE in risk stratification, especially among patients with preserved LVEF.

### Perspectives

Our findings highlight the value of a multiparametric echocardiographic approach in patients with ATTRwt-CM. Although LVEF remains an important prognostic parameter, it is relatively insensitive to early disease and typically becomes impaired only in more advanced stages. This may explain the lack of prognostic discrimination between the mrLVEF and rLVEF groups, as both likely represent an advanced disease stage. Identifying earlier markers of functional decline is therefore essential, particularly given that disease-modifying therapies are most effective in early stages [26].

Measures such as SVi and LV-GLS capture aspects of LV systolic performance that deteriorate earlier than LVEF, enabling earlier detection of disease progression. LV-GLS also provides essential prognostic information and may enhance risk stratification. In addition, TAPSE emerged as a strong independent prognostic marker and improved risk assessment, particularly among patients with pLVEF. These parameters are readily obtainable in routine echocardiography, often through automated analysis and with minimal additional effort.

Incorporating LV-GLS, SVi, and TAPSE into standard echocardiographic evaluation may therefore help identify patients at higher risk who could benefit from closer follow-up and earlier recognition of disease progression.

### Limitations

This study has several limitations. First, its single-center, observational design may limit the generalizability of the findings to broader populations. Second, as with all observational research, the possibility of selection bias cannot be excluded. Third, approximately one-fourth of the patients were enrolled in clinical placebo-controlled trials exploring the effects of new treatments during the follow-up period, which should be considered when comparing outcomes with other ATTRwt-CM cohorts. Finally, despite adjustment for key clinical confounders, residual or unmeasured confounding cannot be ruled out.

## Conclusions

LV and RV systolic dysfunction are frequently present at the time of ATTRwt-CM diagnosis and range across a broad spectrum of severity. LV-GLS and SVi are frequently impaired despite preserved LVEF, highlighting the need for a multiparametric assessment of LV function. Incorporating TAPSE for RV evaluation may further refine risk stratification, particularly when LVEF is preserved.

## Data Availability

Restrictions apply to the availability of the data that supports the findings of this study. The data were accessed under license from the Regional Research Office of the Central Denmark Region (Region Midtjylland) for the current study and are therefore not publicly available. Data are, however, available from the authors upon reasonable request and with permission from the Central Denmark Region.

## Abbreviations

ATTR-CM: Transthyretin amyloid cardiomyopathy
ATTRwt-CM: Wild-type transthyretin amyloid cardiomyopathy
LAVi: Left atrial volume index
LVEF: Left ventricular ejection fraction
pLVEF: Preserved left ventricular ejection fraction (≥ 50%)
mrLVEF: Mildly reduced left ventricular ejection fraction (40–49%)
rLVEF: Reduced left ventricular ejection fraction (< 40%)
LV-GLS: Left ventricular global longitudinal strain
NAC: National Amyloidosis Centre staging system
RAVi: Right atrial volume index
SVi: Stroke volume index
TAPSE: Tricuspid annular plane systolic excursion
